# High-Protein, Low-Glycaemic Meal Replacement Decreases Fasting Insulin and Inflammation Markers—A 12-Month Subanalysis of the ACOORH Trial

**DOI:** 10.3390/nu13051433

**Published:** 2021-04-23

**Authors:** Kerstin Kempf, Martin Röhling, Winfried Banzer, Klaus Michael Braumann, Martin Halle, David McCarthy, Hans Georg Predel, Isabelle Schenkenberger, Susanne Tan, Hermann Toplak, Aloys Berg, Stephan Martin, on behalf of ACOORH Study Group

**Affiliations:** 1 West-German Center of Diabetes and Health, Düsseldorf Catholic Hospital Group, 40591 Düsseldorf, Germany; martin.roehling@vkkd-kliniken.de (M.R.); stephan.martin@vkkd-kliniken.de (S.M.); 2 Department of Sports Medicine, Institute for Sports and Sport Science, University of Frankfurt, 60487 Frankfurt, Germany; banzer@sport.uni-frankfurt.de; 3 Department of Sports and Movement Medicine, Faculty of Psychology and Human Movement Sciences, University of Hamburg, 20148 Hamburg, Germany; braumann@uni-hamburg.de; 4 Department of Prevention, Rehabilitation and Sports Medicine, Klinikum rechts der Isar, Technical University of Munich (TUM), 80992 Munich, Germany; martin.halle@mri.tum.de; 5 DZHK (German Centre for Cardiovascular Research), Partner Site Munich Heart Alliance, 80802 Munich, Germany; 6 Public Health Nutrition Research Group, London Metropolitan University, London N7 8DB, UK; d.mccarthy@londonmet.ac.uk; 7 Institute of Cardiovascular Research and Sports Medicine, German Sport University Cologne, 50933 Cologne, Germany; predel@dshs-koeln.de; 8 KARDIOS, Cardiologists in Berlin, 10787 Berlin, Germany; schenkenberger@klinische-forschung-berlin.de; 9 Department of Endocrinology, Diabetes and Metabolism and Division of Laboratory Research, University Hospital Essen, University Duisburg-Essen, 45122 Essen, Germany; susanne.tan@uk-essen.de; 10 Department of Medicine, Division of Endocrinology, Medical University of Graz, 8010 Graz, Austria; hermann.toplak@medunigraz.at; 11 Faculty of Medicine, University of Freiburg, 79117 Freiburg, Germany; berg.aloys@web.de; 12 Faculty of Medicine, Heinrich-Heine-University Düsseldorf, 40591 Düsseldorf, Germany

**Keywords:** fasting insulin, lifestyle intervention, protein-rich, low-glycaemic meal replacement, low-carbohydrate, overweight, obesity, weight reduction, multicentre study, RCT

## Abstract

Lifestyle interventions, including meal replacement, are effective in the prevention and treatment of type-2-diabetes and obesity. Since insulin is the key weight regulator, we hypothesised that the addition of meal replacement to a lifestyle intervention reduces insulin levels more effectively than lifestyle intervention alone. In the international multicentre randomised controlled ACOORH (Almased Concept against Overweight and Obesity and Related Health Risk) trial, overweight or obese persons who meet the criteria for metabolic syndrome (*n* = 463) were randomised into two groups. Both groups received nutritional advice focusing on carbohydrate restriction and the use of telemonitoring devices. The intervention group substituted all three main meals per day in week 1, two meals per day in weeks 2–4, and one meal per day in weeks 5–26 with a protein-rich, low-glycaemic meal replacement. Data were collected at baseline and after 1, 3, 6 and 12 months. All datasets providing insulin data (*n* = 446) were included in this predefined subanalysis. Significantly higher reductions in insulin (−3.3 ± 8.7 µU/mL vs. −1.6 ± 9.8 µU/mL), weight (−6.1 ± 5.2 kg vs. −3.2 ± 4.6 kg), and inflammation markers were observed in the intervention group. Insulin reduction correlated with weight reduction and the highest amount of weight loss (−7.6 ± 4.9 kg) was observed in those participants with an insulin decrease > 2 µU/mL. These results underline the potential for meal replacement-based lifestyle interventions in diabetes prevention, and measurement of insulin levels may serve as an indicator for adherence to carbohydrate restriction.

## 1. Introduction

Weight gain or loss is regulated by the anabolic hormone insulin [1]. It is a key regulator for not only promoting glucose uptake and lipogenesis but also inhibiting lipolysis [2]. Even brief increases in food consumption lead to immediate increases in insulin levels [3], and permanently elevated insulin levels have been shown to be associated with weight gain and obesity [1]. Moreover, insulin is involved in the regulatory processes of immune cells promoting subclinical inflammation [4,5], a further independent risk factor for type-2-diabetes.

It is well known that lifestyle interventions have been successful not only in the prevention [6,7,8] but also in the treatment of type-2-diabetes, even reaching diabetes remission [9,10]. Especially in studies demonstrating remission, an energy-restricted diet led to a rapid drop in insulin levels and restoration of biphasic glucose-induced insulin release [11]. Nevertheless, lifestyle interventions are often criticised for being unsustainable because a substantial number of participants are not able to adhere to the food restrictions during studies or old eating habits, such as a more frequent carbohydrate consumption, return after the end of interventions.

The Almased Concept against Overweight and Obesity and Related Health Risk (ACOORH) trial [12,13,14] is an international multicentre randomised controlled intervention study comparing the effects of a meal replacement-based lifestyle intervention vs. lifestyle intervention alone in overweight or obese adults with risk factors for metabolic syndrome. Previously published data demonstrated significantly higher success in weight reduction in the meal replacement intervention group compared to the control group with an estimated treatment difference (ETD) −3.2 kg (−4.0; −2.5) (*p* < 0.001) [12]. Moreover, a subanalysis of participants with prediabetes at baseline showed that reconversion to normoglycemia was significantly more often achieved in the intervention group (50% vs. 31%; *p* < 0.05) [13].

So far, it is unclear to what extent carbohydrate reduction achieved by protein-rich, low-glycaemic meal replacement affects the insulin level in overweight or obese people with risk factors for metabolic syndrome. Therefore, in this subanalysis of the ACOORH trial, we examined the effect of the meal replacement intervention on the change in insulin levels in relation to weight loss and inflammation markers with respect to participants’ adherence to the intervention.

## 2. Materials and Methods

### 2.1. Study Design and Population

**Figure 1:**
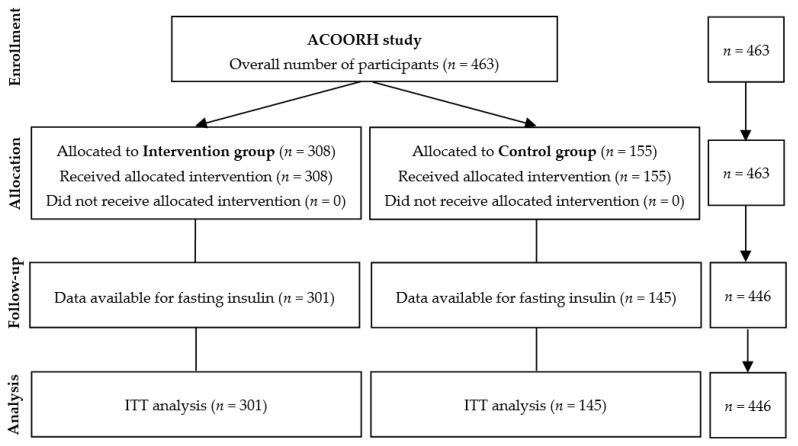
Flow chart.

From 463 participants of the initial ACOORH trial cohort, only those who had a complete set of data regarding fasting insulin (*n* = 446) were considered in the present subanalysis. Individuals (*n* = 17) without fasting insulin values were excluded from this analysis (Figure 1). Details of the international multicentre ACOORH intervention had been previously published [12,13,14]. In brief, participants were randomised with a 1:2 allocation ratio into either a lifestyle intervention control group (*n* = 155) or a meal replacement-based lifestyle intervention group (*n* = 308). Twenty-six weeks of an intensive lifestyle intervention were followed by a moderate intensive follow-up phase until week 52. The first participant was included in January 2015 and the last examination took place in August 2017. Individuals 21–65 years old with a body mass index (BMI) of 27–35 kg/m^2^ and/or a waist circumference of ≥ 88 or ≥ 102 cm (females and males, respectively), and at least one of the following criteria of metabolic syndrome: (a) fasting blood glucose (FBG) 100–125 mg/dL, (b) triglycerides 150–400 mg/dL, (c) high-density lipoprotein (HDL) cholesterol < 40 mg/dL, or (d) untreated systolic blood pressure of 140–160 mmHg or diastolic blood pressure of 90–100 mmHg or use of antihypertensive medication, were eligible for participation in the ACOORH trial. Participants were excluded when one or more of the following exclusion criteria existed: (i) diabetes mellitus with FBG ≥ 126 mg/dL or HbA1c ≥ 6.5% (≥ 48 mmol/mol) or diabetes-related medical history (e.g., antidiabetic drugs or medical records); (ii) total body weight > 141 kg; (iii) acute infections; (iv) chronic diseases, such as cancer, asthma, chronic obstructive pulmonary disease, chronic gut diseases, liver cirrhosis, nephropathy, and kidney insufficiency with glomerular filtration rate < 30 mL/min/1.73 m^2^, dementia, or psychoses; (v) plans to move to areas not served by ACOORH; (vi) (planned) smoking cessation during the study; (vii) use of medication for active weight reduction; (viii) pregnancy or breast feeding; and (ix) known intolerance with components of the used meal replacement.

### 2.2. Intervention and Meal Replacement Regimen

Participants of both groups visited the study centre at baseline as well as after 4, 12, 26, and 52 weeks. Participants received nutrition counselling at the study visits and were instructed to increase physical activity [12]. Additionally, both groups were equipped with telemetric scales and pedometers that automatically transferred data into a personalised online portal (for details see [12]). During study visits, acquired data (e.g., steps, weight, diet protocols) were discussed and participants were motivated to achieve their individual goals (e.g., weight reduction, healthy lifestyle changes).

Participants of the intervention group additionally received a high-protein, low-glycaemic meal replacement (Almased, Almased Wellness GmbH, Oberding, Germany) during the first 26 weeks as previously described [12]. In brief, in the first week all three main meals were replaced, then in weeks 2–4 only breakfast and dinner, and afterward only dinner was replaced until week 26. An accompanying manual included information about the preparation of the meal replacement as well as general facts about low-carbohydrate meals and their influence on blood glucose and insulin levels, hunger, and weight loss.

### 2.3. Outcomes and Measurements

Anthropometrical, clinical (weight, BMI), and laboratory data (fasting insulin, fasting blood glucose, HbA1c) were measured as previously described [12,13,14] at baseline; after 4, 12, and 26 weeks of intervention; and after 52 weeks. C-reactive protein (CRP) and interleukin (IL)-6 were analysed in an accredited medical laboratory (Synlab, Leinfelden, Germany). Adverse and serious adverse events were continuously reviewed by an external monitor [12]. The assessors were blinded to group allocation.

### 2.4. Statistics

Sample-size calculation and its assumptions can be found elsewhere [12]. Per-protocol (PP) and intention-to-treat (ITT) analyses were performed, although, if not otherwise stated, the ITT results were reported. The last observation carried forward (LOCF) principle was used for imputation of missing values. The present predefined subanalysis focuses on the tertiary outcome of within-group changes from baseline to week 12 and week 52 regarding fasting blood insulin and the accompanied parameters, such as weight, BMI, HbA1c, and fasting blood glucose. In order to analyse the influence of fasting insulin levels on weight changes, tertile stratification was performed for the achieved insulin reductions after 6 months (1st tertile = insulin reduction of > 2 µU/mL; 2nd tertile = constant insulin values with changes ≤ 2 µU/mL; 3rd tertile = insulin increase > 2 µU/mL) and was related to the weight reduction at the same time points. These changes were compared between control and intervention groups. In addition, the weight courses of the intervention group in these three tertiles were analysed. In a further subanalysis, we separately analysed data of those participants who were compliant with the study protocol, i.e., the participants who performed the meal replacement as recommended and completed all study visits. They were defined as “completers”. Those who either stopped the meal replacement prematurely or who did no longer appear for the visits were defined as “dropouts”.

Non-parametric data were analysed with Mann-Whitney U, Wilcoxon, or Kruskal-Wallis tests along with Dunn’s multiple comparison test. Parametric data were evaluated with Student’s *t*-test, paired *t*-test, or analysis of variance with repeated measures. Multivariable linear regression analyses were performed to examine the associations of changes in fasting insulin levels and weight after 4, 12, 26, and 52 weeks of intervention and were corrected for baseline values. All statistical tests were two-sided, and the level of significance was set at *p* = 0.05. The statistical analysis was performed by an independent institute (ACOMED statistik^®^, Leipzig, Germany) not involved in the study execution. All analyses were performed using SPSS 22.0 (SPSS Inc., Chicago, IL, USA) and GraphPad Prism 6.04 (GraphPad Software, San Diego, CA, USA).

## 3. Results

### 3.1. Stronger Improvement in Fasting Insulin Levels and Body Weight in the Intervention Group

Baseline characteristics (Table 1) did not differ significantly between the control and intervention groups.

**Figure 2:**
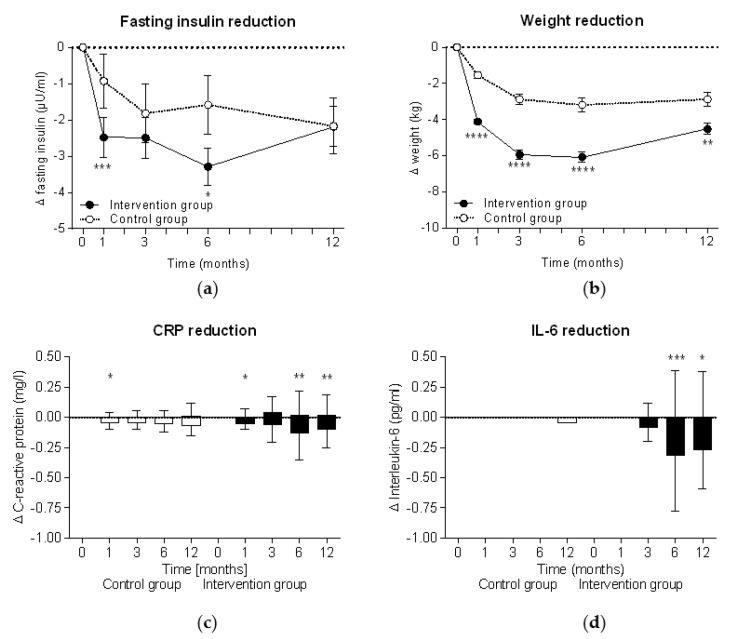
Intention-to-treat analyses of improvements during the study. Mean ± standard errors of changes in (**a**) fasting insulin levels and (**b**) body weight are shown and the Mann-Whitney test was used for intergroup analyses of the intervention group (*n* = 301) and the control group (*n* = 145). Tukey plots with median ± interquartile range are shown for (**c**) C-reactive protein (CRP) and (**d**) interleukin 6 (IL-6). Wilcoxon signed rank test was used for intragroup analyses (*, *p* < 0.05; **, *p* < 0.01; ***, *p* < 0.001; ****, *p* < 0.0001).

During the intervention, fasting insulin levels significantly decreased in both groups (within group comparison: *p* < 0.0001 at all time points in the intervention group and *p* < 0.05 in the control group), although insulin reduction was significantly higher in the intervention group (Figure 2a). In parallel, significant weight reduction was observed in both groups (within group comparisons for both: *p* < 0.0001 at all time points) but also showed a higher reduction in the intervention group (Figure 2b). The highest insulin level (−3.3 ± 8.7 µU/mL in the intervention group vs −1.6 ± 9.8 µU/mL in the control group) and weight reductions (−6.1 ± 5.2 kg vs. −3.2 ± 4.6 kg) were observed after six months at the end of the intervention phase. In cases where the meal replacement was discontinued in accordance with the study protocol at week 26, insulin levels in the intervention group started to increase and reached the insulin levels of the control group. Accordingly, the weight data also show a re-increase after week 26 when the meal replacement phase was finished.

### 3.2. Stronger Improvement in Chronic Inflammation in the Intervention Group

There was a trend toward a reduction of the proinflammatory inflammation markers CRP and IL-6 in the intervention group following the intervention, peaking after six months, while the control group showed no consistent change over time (Figure 2c,d).

### 3.3. Correlation of Improvements in Fasting Insulin Reduction and Weight Loss

A significant correlation between the reduction of fasting insulin and body weight could be observed consequently at all observation times throughout the study (Table 2).

**Figure 3:**
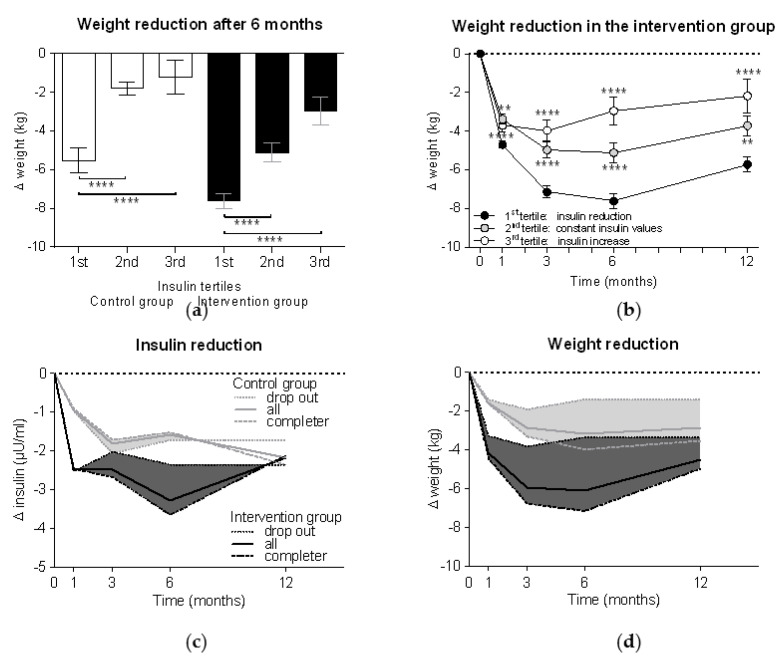
Weight reduction is related to insulin reduction. Participants were divided into three tertile groups according to their changes in insulin levels after six months compared to the baseline (1st tertile = insulin reduction of > 2 µU/mL; 2nd tertile = constant insulin values with changes ≤ 2 µU/mL; 3rd tertile = insulin increase > 2 µU/mL). Weight reduction (**a**) after six months in both groups and (**b**) in the intervention group alone (*n* = 301) during the whole study period was compared between tertile groups using Kruskal-Wallis test with Dunn’s multiple comparison test (**, *p* < 0.01; ****, *p* < 0.0001). Subanalyses of reduction in (**c**) insulin levels and (**d**) weight were performed for dropouts (*n* = 44 in the control group and *n* = 85 in the intervention group) and completers (*n* = 101 in the control group and *n* = 216 in the intervention group). The areas between the curves of the dropouts (dotted lines) and the completers (dashed lines) were filled with colour and the curves of the whole group (solid lines; here described with “all”) were added.

Tertile analyses demonstrated in both control and intervention groups significantly higher weight reductions in the 1st insulin tertile compared to the 2nd and 3rd tertiles (*p* < 0.0001 each) (Figure 3a). Thus, those participants who could reduce their insulin levels more than 2 µU/mL had a weight loss −7.6 ± 4.9 kg in the intervention group vs. −5.5 ± 4.9 kg in the control group (*p* < 0.01). Participants with unchanged insulin levels demonstrated a weight reduction 5.1 ± 5.0 kg in the intervention group vs. 1.8 ± 1.4 kg in the control group (*p* < 0.0001), while increased insulin levels were associated with weight loss of 3.0 ± 4.9 kg in the intervention group vs. 1.2 ± 5.0 kg in the control group. The tertile analyses further demonstrated that the participants with insulin reduction also achieved the highest weight reduction over the course of the study, while the group with increased insulin values already started to regain weight after six months (*p* < 0.0001 vs. the 1st tertile as control) (Figure 3b).

### 3.4. Dropouts Explain the Re-Increase in Insulin and Weight

In order to better understand how insulin levels are associated with weight loss, we performed a subanalysis based on adherence to the study protocol (i.e., meal replacement completers vs. dropouts). Figure 3c shows that participants in the intervention group who achieved a mean reduction in insulin levels of 2.5 ± 9.5 µU/mL in the first four weeks of the study, insulin levels rose again when they stopped meal replacement, while the completers achieved insulin reductions of −3.7 ± 8.6 µU/mL. The same effects can be seen with weight, but with a certain delay (Figure 3d). When comparing both groups, we can see that the dropouts of the intervention group from week 12 onward achieved a weight reduction slightly higher than the completers of the control group.

## 4. Discussion

The international multicentre randomised controlled ACOORH trial demonstrated the superiority of a lifestyle intervention accompanied by a dietary change of a high-protein, low-glycaemic meal replacement compared to a control lifestyle intervention alone. Consequently, this superiority led to a greater reduction in fasting insulin levels. Furthermore, the insulin reduction correlated with the achieved weight reduction and was accompanied by improvements in inflammation markers. Participants who prematurely ended meal replacement still achieved insulin and weight improvements comparable to the control group. After the end of the intervention, both insulin levels and weight increased again but remained significantly below baseline levels.

In this subanalysis of the ACOORH trial, we primarily focused on fasting insulin as insulin not only mediates glucose uptake from the blood into the cells but also has further physiological regulatory functions. For example, insulin inhibits lipolysis at a much lower concentration than it is needed for glucose uptake [1]. By using a microdialysis technique in combination with a three-step hyperinsulinaemic glucose clamp, Jacob et al. [2] demonstrated in nineteen lean, healthy subjects that low physiological concentrations of insulin are able to inhibit lipolysis in muscle up to nearly 50% and in adipose tissue up to 75%. Thus, every insulin-releasing carbohydrate consumption might be able to slow down or even completely block lipolysis in the human body. To what degree the lipolysis is inhibited depends on the BMI. When lean people (BMI < 25 kg/m^2^) consumed 75 g glucose, they show only a small and short-term increase in insulin [15]. However, when people with obesity (BMI > 30 kg/m^2^) ingested the same amount of glucose, their insulin levels rose for hours. In fact, in participants with obesity, insulin levels were already increased in the fasting state, rose nearly twice as high after glucose consumption, and remained elevated for nearly one hour longer compared to lean individuals, resulting in an inhibition of lipolysis. When obese people consumed carbohydrate-containing meals and snacks throughout the day, the inhibitory activity of insulin on lipolysis would explain why losing weight is hardly achievable in the obese state. If obese people want to reduce weight, the first aim should be to lower their insulin levels [16,17]. Therefore, in the first phase of a diary intervention it is necessary to strictly reduce or nearly eliminate carbohydrate intake [18,19]. Furthermore, food intake needs to be reduced to a maximum of three meals per day so that insulin levels can decrease between each meal and lipolysis can be activated. Meal frequency is a controversial topic, however, the evidence in favour of a lower meal frequency was demonstrated in a recently published review [20]. Reduced meal frequency with 2–3 meals per day and regular fasting periods were shown to provide physiological benefits [21,22]. Analyses of isocaloric diets of either two or six meals per day on energy expenditure, measured in a metabolic chamber, showed a significantly higher energy expenditure at night with a two-meal diet [23]. Thus, we instructed our participants to reduce their carbohydrate intake and to eat no more than three meals per day. Participants who achieved the greatest success in losing weight were those who lowered their insulin levels the most. Therefore, measuring insulin levels could possibly be used in the future to monitor the degree of compliance with the carbohydrate restriction.

It is well known that metabolic disorders with elevated insulin levels, such as obesity, metabolic syndrome, and type-2-diabetes, are associated with and accompanied by chronic subclinical inflammation. Thus, insulin is also thought to be involved in regulating the activation status of immune cells. Normally, naive T cells gain energy by oxidation of fatty acids [4]. However, their signal to become activated is conveyed by glucose admission and a switch to aerobic glycolysis [5,24]. Activation with lipopolysaccharide (LPS) also leads to the use of glycolysis in classic proinflammatory macrophages and dendritic cells [25,26]. Another example are IL-4-induced alternatively activated macrophages, which help to suppress inflammatory signals as they become down regulated in hyperinsulinaemia and obesity [27,28,29]. As glycolysis is mainly found in inflammatory and rapidly proliferating immune cells, and in contrast long-living and anti-inflammatory cells are related to β-oxidation, it can be concluded that key enzymes and metabolic programs can instruct immune cells to carry out proinflammatory or anti-inflammatory functions. This relation could explain why increased inflammatory reactions are observed in the context of overeating with a large proportion of carbohydrates. An increased fat metabolism for immune functions, on the other hand, indicates a pronounced anti-inflammatory effect.

Various studies, in which carbohydrate restriction and intermittent fasting were part of the intervention, not only showed a significant reduction in body weight in participants who were overweight but also a concomitant decrease in the concentration of inflammatory markers in the blood [30,31,32]. Missing insulin signalling during fasting can therefore be seen as a regulator of the immune system as it influences the release of inflammatory cytokines, such as IL-6, in the body [33,34,35]. Moreover, intermittent fasting can delay immune senescence, which is characterised by a progressive decline in immune function with increasing age, according to a publication in which the number of hematopoietic stem cells increased fivefold through a fast-imitating diet [36]. Further major changes as a result of nutrition restriction and lowered insulin levels in metabolic pathways and cellular processes, such as lipolysis, autophagy, and increased lifespan, have been discussed in previous reviews [1,37]. Thus, a metabolic change caused by fasting can potentially be as medically effective as approved drugs [38,39,40].

Although the application of meal replacement is still a controversial topic, previous work [12,41] and reviews concerning such intervention studies have shown that adding meal replacement to lifestyle interventions can lead to greater weight reductions [42]. Since it has been shown that an effective change in lifestyle and diet can only be successful in the long-term with intensive support [43], an intervention should be started with high-protein, low-glycaemic meal replacement for the diet change. The advantage here is that the meal composition is clear and easy to use while also containing all the necessary nutrients, vitamins, minerals, and trace elements. In addition, it has been shown that meal replacement can actively reduce insulin levels and these effects can be already seen after one week. Through meal replacement the daily insulin demand could be reduced by 40% in insulin-treated type-2-diabetes patients [44], while in noninsulin-treated type-2-diabetes patients fasting insulin levels reduced by more than half [11]. Concomitantly, Lim et al. [11] demonstrated that the inhibited second-phase insulin secretion is restored after a successful meal replacement-based intervention, indicating that carbohydrate restriction by meal replacement is able to return insulin secretion back to physiological levels. Similar fast effects could only be seen after Roux-en-Y gastric bypass surgery where diabetes remission was accompanied by the normalisation of fasting insulin levels within a few days before significant weight loss occurred and although patients were still obese [45].

If only the ITT analysis is considered, the present data must be viewed with reservations. Since it contains the data of the participants who adhered to the study protocol, as well as those who already finished the meal replacement after four weeks, there is the possibility of an underestimation of the results. The actual effects are therefore better reflected by the completers analysis, as it shows which effects can be achieved when the meal replacement is applied according to the study protocol. The following also applies in any study: those who do not change their lifestyle cannot expect any improvement in metabolic values. However, the data show that even a four-week use of a meal replacement leads to a weight loss comparable to that achieved by the completers of the control group.

A further limitation of the study is that after six months the participants were no longer intensively supported. By stopping the meal replacement and returning to normal eating habits with meals composed of 20 g/day higher carbohydrate content than the meal replacement (carbohydrate consumption in the intervention group was 198 ± 71 g/day at baseline, 146 ± 83 g/day after 12 weeks and 170 ± 95 after 52 weeks [14]) an increase in insulin levels was observed, which in turn was associated with a weight re-gain of approximately 1 kg over six months. Prospectively, the increasing carbohydrate consumption and a subsequent weight gain might support a re-alteration of energy production in immune cells, i.e., from fat oxidation to glycolysis. Consequently, this might lead to a re-increase in inflammation markers and would confirm the regulatory role of insulin in subclinical inflammation. In further studies it would therefore be interesting to examine the effect of re-increasing carbohydrate consumption on the course of CRP and IL-6 and other inflammatory markers.

## 5. Conclusions

In sum, high-protein, low-glycaemic meal replacement-based lifestyle interventions can lead to a greater reduction in insulin levels even if the meal replacement is only used for a short time. The insulin reduction correlated with the achieved weight reduction and was accompanied by an decrease in inflammation markers. Meal replacement-based lifestyle interventions should therefore not only be used in the treatment of manifested type-2-diabetes but also in primary prevention, while insulin measurements might be used to monitor for compliance in carbohydrate restriction.

## Figures and Tables

**Table 1:** Baseline characteristics.

Parameters	Control Group (*n* = 145)	Intervention Group (*n* = 301)
Sex (male/female) (*n*)	57/88 (39%/61%)	99/202 (33%/67%)
Age (years)	50 ± 10	51 ± 10
Fasting insulin (µU/mL)	14.1 ± 9.5	15.3 ± 10.2
Weight (kg)	94 ± 11	92 ± 13
Body Mass Index (kg/m^2^)	31.4 ± 2.3	31.6 ± 2.3
Fasting blood glucose (mg/dL)	94 ± 11	94 ± 13
HOMA-IR	3.3 ± 2.4	3.6 ± 2.5
HbA1c (%)	5.5 ± 0.4	5.5 ± 0.6
Interleukin 6 (pg/mL)	3.2 ± 3.5	3.4 ± 3.6
C-reactive protein (mg/dL)	0.5 ± 1.0	0.5 ± 1.0

Shown are means ± standard deviations, or percentages. In the control group (*n* = 35) and in the intervention group (*n* = 73) datasets were missing for interleukin 6 and C-reactive protein. HOMA-IR, homeostasis model assessment of insulin resistance; HbA1c, glycosylated haemoglobin A1c.

**Table 2:** Associations between Δ fasting insulin and Δ weight.

Parameters	Month	r	*p*	ß	*p*
**Control Group** **(*n* = 145)**	1	0.074	**0.375**	0.086	**0.182**
	3	0.127	**0.128**	0.209	**0.002**
	6	0.294	**<0.001**	0.342	<**0.001**
	12	0.216	**0.009**	0.263	<**0.001**
**Intervention Group** **(*n* = 301)**	1	0.180	**0.002**	0.180	<**0.001**
	3	0.214	**<0.001**	0.195	<**0.001**
	6	0.279	**<0.001**	0.280	<**0.001**
	12	0.208	**<0.001**	0.278	<**0.001**

Bold *p*-values represent significance. Multivariable linear regression analyses were carried out to investigate associations between changes in fasting insulin and weight after 1, 3, 6, and 12 months and were adjusted for baseline values.

## Data Availability

The data presented in this study are available on reasonable request from the corresponding author.
